# Module Based Differential Coexpression Analysis Method for Type 2 Diabetes

**DOI:** 10.1155/2015/836929

**Published:** 2015-08-03

**Authors:** Lin Yuan, Chun-Hou Zheng, Jun-Feng Xia, De-Shuang Huang

**Affiliations:** ^1^School of Electronics and Information Engineering, Tongji University, Shanghai 201804, China; ^2^College of Electrical Engineering and Automation, Anhui University, Hefei 230601, China; ^3^Institute of Health Sciences, Anhui University, Hefei 230601, China

## Abstract

More and more studies have shown that many complex diseases are contributed jointly by alterations of numerous genes. Genes often coordinate together as a functional biological pathway or network and are highly correlated. Differential coexpression analysis, as a more comprehensive technique to the differential expression analysis, was raised to research gene regulatory networks and biological pathways of phenotypic changes through measuring gene correlation changes between disease and normal conditions. In this paper, we propose a gene differential coexpression analysis algorithm in the level of gene sets and apply the algorithm to a publicly available type 2 diabetes (T2D) expression dataset. Firstly, we calculate coexpression biweight midcorrelation coefficients between all gene pairs. Then, we select informative correlation pairs using the “differential coexpression threshold” strategy. Finally, we identify the differential coexpression gene modules using maximum clique concept and *k*-clique algorithm. We apply the proposed differential coexpression analysis method on simulated data and T2D data. Two differential coexpression gene modules about T2D were detected, which should be useful for exploring the biological function of the related genes.

## 1. Introduction

DNA microarray has been widely used as measurement tools in gene expression data analysis [1–4]. Gene expression profiling data from DNA microarray can detect the expression levels of thousands of genes simultaneously, providing an effective way for mining disease-related genes and revealing information of the regulatory networks and biological pathways of genes. Currently, the analysis of gene expression data can be divided into three levels: first, analysis of the expression level of individual genes, determining its function based on gene expression level changes under different experimental conditions: for example, the tumor type specific genes are identified according to the significance of difference in gene expression using the statistical hypothesis testing analysis method; second, study of gene interaction and coregulation through the combination of genes and grouping; and, third, an attempt to deduce the potential gene regulatory networks mechanism and explain the observed gene expression data.

Among the microarray data analysis methods, gene differential expression analysis is one of the most widely used types of analysis for disease research. Gene differential expression analysis method selects differentially expressed genes according to expression change value of a single gene. In fact, gene expression value change between normal samples and disease samples can be used to present the possibility of the relation between gene and disease. However, the traditional pathogenicity genes selection methods based on gene expression data treat each gene individually and interaction between them is not considered. Actually, genes and their protein products do not perform their functions in isolation [5, 6], but in cooperation. Functional changes such as alteration in tumor cell growth process, energy metabolism, and immune activity were accompanied with coexpression changes. Differentially expressed genes selection methods often focus only on the size of the single genes and the relationship of individual genes and disease, ignoring a plurality of pathogenic genes of the complex disease as a gene module with disease related, as well as within the module gene [7].

Differential coexpression analysis, as a more comprehensive technique to the differential expression analysis, was raised to research gene regulatory networks and biological pathways of phenotypic changes through measure gene correlation changes between disease and normal conditions. Differential coexpression genes are defined as genes whose correlated expression pattern differs between classes [8]. The gene coexpression changes between different conditions indicate gene regulatory pathways and networks associated with disease. In gene differential coexpression analysis, a pair of gene expression datasets under disease and normal conditions is transformed to a pair of coexpression matrix in which links represent transcriptionally correlated gene pairs [5]. Until now, methods for differential coexpression analysis of gene expression data have been extensively researched, and multiple algorithms have been developed and tested [9–12]. In those gene differential coexpression analysis methods, the most common choice of similarity measurement is Pearson's correlation coefficients. However, Pearson's correlation is sensitive to outliers. So biweight midcorrelation (bicor) is considered to be a good alternative to Pearson's correlation since it is more robust to outliers [13].

In biomedical research, many complex diseases are contributed jointly by alterations of numerous genes; they often coordinate together as a functional biological pathway or network and are highly correlated. With recent interest of gene differential coexpression analysis in the gene network or module, gene module analysis has emerged as a novel holistic approach for microarray analysis. Somewhat large units, made up of genes, are more densely connected to each other than to the rest of the network, are often referred to as modules, and have been considered to be the essential structural units of real gene networks. There exists overlap among gene modules in large real networks.

Until now, there are many methods to find gene modules. For example, Butte and Kohane [14] proposed a systems-based approach called Entropy Minimization and Boolean Parsimony (EMBP) that identifies, directly from gene expression data, modules of genes that are jointly associated with disease. Kostka and Spang [15] used additive model to find differential coexpression gene modules. Prieto et al. [16] used altered expression based on improved additive model, optimal residual ratio, and minimum* F*-distribution to find differential coexpression gene modules. However, the microarray data contains a large number of genes; those methods need to search all gene expression data resulting in a large amount of computation; the process is very time-consuming even using optimized search algorithm.

The maximum clique analysis can avoid exhaustive search and quickly find maximum gene module with biological significance. The maximum clique problem (MCP) is a classical combinatorial optimization problem in graph theory. In 1957, Ross and Harary [17] first proposed the deterministic algorithm to solve the maximum clique problem. Since then some researchers had presented a variety of algorithms to solve this problem. The maximum clique problem is widely used in different areas, such as signal transmission, computer vision, and biological research. In this study, a gene coexpression network can be treated as a graph; gene is represented by vertex and coexpression relationship is represented by edge. We will use* k*-clique algorithm [18], which is an effective and deterministic method for uniquely identifying overlapping modules in large real networks. We first show some basic definitions.* k*-cliques, the central objects of* k*-clique algorithm investigation, are defined as complete (fully connected) subgraphs of *k* vertices.* k*-clique adjacency is as follows: two* k*-cliques are adjacent if they share some vertices.* k*-clique chain is as follows: a subgraph, which is the union of a sequence of adjacent* k*-cliques. We use* k*-clique algorithm to find gene cliques, and maximum clique concept is used to quickly find large gene modules which are made of* k*-clique chain. For the sake of convenience, we use the terms graph and community or network interchangeably, the former stressing the mathematical concept and the latter the application.

In this paper, we proposed a new approach for gene differential coexpression analysis in gene modules level based on combining biweight midcorrelation, differential coexpression threshold strategy, and maximum clique concept and* k*-clique analysis. Biweight midcorrelation measures the coexpression relationship between genes and the* k*-clique analysis with maximum clique concept quickly finds maximum disease-related module with biological significance. We use the approach to further investigate the gene module in order to gain insight into coexpression relationship between genes. The algorithm can find differential coexpression disease genes modules and global coexpression patterns are determined for type 2 diabetes expression dataset. As far as we know, no one has done this experiment.

The rest of the paper is organized as follows.  Section 2 describes the methods proposed in this study. The biweight midcorrelation coefficients, “gene differential coexpression threshold” strategy, and threshold selection strategy are first presented, and the algorithm of* k*-clique is consequently given.  Section 3 presents the experiment on simulated data and type 2 diabetes (T2D) in rats dataset.  Section 4 concludes the paper and outlines directions of future work.

## 2. Methods

### 2.1. Biweight Midcorrelation for Differential Coexpression

Differential coexpression analysis usually requires the definition of “distance” or “similarity” between measured datasets, the most common choice being Pearson's correlation coefficients. However, Pearson's correlation coefficient is sensitive to outliers [13]. Biweight midcorrelation is considered to be a good alternative to Pearson's correlation since it is more robust to outliers. Example of a gene expression matrix is as follows:

(1) $$\begin{array}{c} \left[\begin{array}{ccccc} & Gene1 & Gene2 & \cdots & Genep\\ Z_{1} & X_{11} & X_{12} & \cdots & X_{1p}\\ Z_{2} & X_{21} & X_{22} & \cdots & X_{2p}\\ \vdots & \vdots & \vdots & \ddots & \vdots \\ Z_{n} & X_{n1} & X_{n2} & \cdots & X_{np} \end{array}\right]. \end{array}$$

For each sample *Z*_*i*_, we measure expression levels for a set of genes, so *X*_*ij*_ is the measurement of the expression level of the *j*th gene for the *i*th sample, where *j* = 1,…, *p*. The *x*th column vector of matrix represents gene expression profile of gene *X*. In order to define the biweight midcorrelation (bicor) [13] of two numeric vectors *x* = (*x*_1_,…, *x*_*m*_) and *y* = (*y*_1_,…, *y*_*m*_), we first define *u*_*i*_, *v*_*i*_ with *i* = 1,…, *m*:

(2) $$\begin{array}{c} u_{i}=\frac{x_{i}-med\left(x\right)}{9mad\left(x\right)},\\ v_{i}=\frac{y_{i}-med\left(y\right)}{9mad\left(y\right)}, \end{array}$$

where med(*x*) is the median of vector *x*, mad(*x*) is the median absolute deviation of vector *x*, mad(*x*) is the median of new numeric vector in which each number is absolute difference between original vector value and med(*x*); this leads us to the definition of mad(*x*) and weight *w*_*i*_ for *x*_*i*_, which are

(3) $$\begin{array}{c} mad\left(x\right)=med\left(\left|x_{i}-med\left(X\right)\right|\right),\\ w_{i}^{\left(x\right)}={\left(1-u_{i}^{2}\right)}^{2}I\left(1-\left|u_{i}\right|\right), \end{array}$$

where the indicator *I*(1 − |*u*_*i*_|) takes 1 if 1 − |*u*_*i*_| > 0 and 0 otherwise. Thus, the weight *w*_*i*_^(*x*)^ is close to 1 if *x*_*i*_ is close to med(*x*), approaches 0 when *x*_*i*_ differs from by nearly  9mad(*x*), and is 0 if *x*_*i*_ differs from med(*x*) by more than 9mad(*x*). An analogous weight *w*_*i*_^(*x*)^ can be defined for *y*_*i*_. Given the weights, we can define biweight midcorrelation of *x* and *y* as

(4) bicorx,y=∑i=1mxi−medxwixyi−medywiy ·∑j=1mxj−medxwjx2hhhih·∑k=1myk−medywky2−1.

It should be noted that the equations of biweight midcorrelation do not involve an explicit identification of outliers, and all elements whose weight *w*_*i*_ = 0 can be considered outliers. The user can also set up the maximum allowed proportion of outliers using the argument “maxPOutliers”; the “maxPOutliers” is interpreted as the maximum proportion of low and high outliers separately. For the value of bicor from −1 to 1, −1 represents the maximum negative correlation and 1 represents the maximum positive correlation. Zero represents irrelevant correlation.

### 2.2. The “Differential Coexpression Threshold” Strategy

We used biweight midcorrelation to measure every pair of genes in the gene expression dataset and get a gene coexpression matrix. The gene coexpression matrix is a square and symmetric matrix *P* whose rows and columns correspond to the genes and whose element *P*_*ij*_ denotes the coexpression relationship between genes *i* and *j*. In this paper, we use *A*_*GN*_ which represents gene coexpression adjacency matrix in normal conditions and *A*_*GD*_ which represents gene coexpression adjacency matrix in disease condition. To find differential coexpression gene modules which are coexpressed in normal condition and not related to disease condition, we set two thresholds *T*_1_ for adjacency matrix *A*_*GN*_ in normal condition and *T*_2_ for adjacency matrix *A*_*GD*_ in disease condition. *A*_*GN*_(*i*, *j*) is set to 1 if value of *A*_*GN*_(*i*, *j*) is greater than or equal to *T*_1_; otherwise, *A*_*GN*_(*i*, *j*) is set to 0 and *A*_*GD*_(*i*, *j*) is set to 1 if value of *A*_*GD*_(*i*, *j*) is less than or equal to *T*_2_; otherwise, *A*_*GD*_(*i*, *j*) is set to 0. We integrated *A*_*GN*_ and *A*_*GD*_ into a matrix *A*_*G*_ after we had intersection of the corresponding elements of *A*_*GN*_ and *A*_*GD*_. *A*_*G*_(*i*, *j*) = 1 means coexpression value of gene *i* and gene *j* in *A*_*GN*_ is greater than or equal to *T*_1_, and coexpression value of genes *i* and *j* in *A*_*GD*_ is less than or equal to *T*_2_. *A*_*GN*_(*i*, *j*) also can be set to 1 if value of *A*_*GN*_(*i*, *j*) is less than or equal to *T*_1_ and *A*_*GD*_(*i*, *j*) is set to 1 if value of *A*_*GD*_(*i*, *j*) is greater than or equal to *T*_2_. The method is shown in (5).

With the above mentioned strategy, we also set *A*_*G*_(*i*, *j*) = 1 if the absolute value of *A*_*GN*_(*i*, *j*) subtracting *A*_*GD*_(*i*, *j*) is greater than or equal to *T*_3_ and the absolute value of *A*_*GN*_(*i*, *j*) is greater than or equal to the absolute value of *A*_*GD*_(*i*, *j*) simultaneously. This is a special type of coexpression change. In reality, coexpression reversal probably has biological significance. The coexpression reversal between normal condition and disease condition has advantage in disease. For example, the coexpression of* p53* and* Klf4* recently reported that the positive or negative correlation between these two genes determines the outcome of DNA damage, DNA repair, or apoptosis [19]. We believe that our attention to this special coexpression change will help to explore subtle mechanisms involved in genes transcriptional regulation. We excavated maximum cliques which have biological significance from *A*_*G*_ adjacency matrix to further investigate gene regulatory networks. Consider the following:

(5) if  AGNi,j≥T1,  then  AGNi,j=1,else  AGNi,j=0;if  AGDi,j≤T2,  then  AGDi,j=1,else  AGDi,j=0;AGi,j=AGNi,j∩AGDi,jif  AGNi,j≤T1,  then  AGNi,j=1,else  AGNi,j=0;if  AGDi,j≥T2,  then  AGDi,j=1,else  AGDi,j=0;AGi,j=AGNi,j∩AGDi,j.

### 2.3. The Threshold Selection Strategy

The two real value adjacency matrixes are transformed into a binary matrix which contains two elements 0 and 1 only. Choosing different thresholds will lead to different results; too large *T*_1_ threshold or too small *T*_2_ threshold will lead to small link number, low density clique, and lost biological significance cliques. On the other hand, too small *T*_1_ or too large *T*_2_ will lead to many overlapping cliques. They are not helpful for finding biological significance differential coexpression gene disease-related modules. In fact, how to choose a reasonable threshold in conversion process is a problem which needs to be further studied. Generally, the selection of the threshold can be based on the proportion of outliers in the figure or the density of graph. The outlier is the point which is not connected to any edges. The density is defined as the ratio of number of edges to the maximum possible number of edges in the graph. The density of clique is 1.

For gene expression data analysis, closely linked functional module is not the strict sense of maximum clique due to the lack of certain section. In this paper, we use density to measure approximation degree of functional module with gene differential coexpression clique, which may be having more biological significance.

### 2.4. The Maximum Clique Concept and **k**-Clique Algorithm

Graph theoretical concepts are useful for the description and analysis of interactions and relationships in biological systems. In gene coexpression graph, gene is represented by vertex and coexpression relationship by edge. *G* = (*V*, *E*) is an arbitrary undirected and weighted graph unless otherwise specified in graph theoretical concepts. *V* = {1, 2,…, *n*} is the vertex set of *G*, and *E* is the edge set of *G*. For each vertex *i* ∈ *V*, a positive weight *w*_*i*_ is associated with *i*. *A*_*G*_ = (*a*_*ij*_)_*n*×*n*_ is the adjacency matrix of *G*, where *a*_*ij*_ = 1 if (*i*, *j*) ∈ *E* is an edge of *G*, and *a*_*ij*_ = 0 if (*i*, *j*) ∉ *E*. Genes and relationship between genes are represented by vertex and edge, respectively.

A graph *G* = (*V*, *E*) is complete if all its vertices are pairwise adjacent; that is, for all *i*, *j* ∈ *V*, (*i*, *j*) ∈ *E*. A clique *C* is a subset of *V* such that *G*(*C*) is complete. The maximum clique problem asks for a clique of maximum weight. An independent set (stable set and vertex packing) is a subset of *V*, whose elements are pairwise nonadjacent. The maximum independent set problem asks for an independent set of maximum cardinality. The size of a maximum independent set is the stability number of *G* (denoted by *α*(*G*)). The maximum weight independent set problem asks for an independent set of maximum weight. A maximum clique means a clique which is a subset of the nodes in *V* in which every pair of nodes in the subset is joined by an edge and is not a proper subset of any other cliques [20].

In application, the identification of maximal cliques is often of limited interest since the requirement of complete connectivity is so restrictive. When dealing with imperfect systems or with experimental data, we may need to consider more general notions of cohesive subgroups. In this paper, we consider different notions of cohesive subgroups that include *n*-cliques, *k*-plexes, and *λ*-sets [18]. It is well known that the nodes of large real networks have a power law degree distribution [21]. Most real networks typically contain parts in which the nodes (units) are more highly connected to each other compared to the rest of the network. The sets of such nodes are usually called clusters, communities, cohesive groups, or modules [22–26], which have no widely accepted unique definition. The basic observation on which our modules definition relies is that a typical gene differential coexpression module consists of several complete (fully connected) subcliques that tend to share many of their nodes. To find meaningful communities, several basic requirements should be satisfied: it cannot be too restrictive, should be based on the density of links, is required to be local, should not yield any cut-node or cut link (whose removal would disjoin the community), and, of course, should allow overlaps. We employ the community definition specified above because none of the others in the literature satisfy all these requirements simultaneously [27–29].

*k*-clique algorithm for detecting gene differential coexpression modules in a network has been published in the paper [26].* k*-clique algorithm is also named clique percolation method. The existing divisive and agglomerative methods recently used for large real networks have some disadvantages. Divisive methods cut the network into smaller and smaller pieces; each node is forced to remain in only one community and be separated from its other communities, most of which then necessarily fall apart and disappear [27, 30]. The agglomerative [31] method has the same problem. The* k*-clique algorithm has demonstrated the advantages over the divisive method and agglomerative method. In the algorithm, although the numerical determination of the full set of* k*-clique communities is a polynomial problem, the algorithm is exponential and significantly more efficient for the graphs corresponding to actual data. The* k*-clique algorithm first locates all cliques (maximal complete subgraphs) of the network and then identifies the communities by carrying out a standard component analysis of the clique-clique overlap matrix [28]. The* k*-clique algorithm uses the threshold probability *d*(*k*) (critical point) of* k*-clique percolation to find all maximal complete subgraphs. The critical point is shown in (6), where *N* is the number of genes or vertex of graph:

(6) $$\begin{array}{c} d\left(k\right)=\frac{1}{{\left[\left(k-1\right)N\right]}^{1/\left(k-1\right)}}. \end{array}$$

The* k*-clique algorithm gives two plausible choices to measure the size of the largest* k*-clique percolation cluster in (7) and (8). The most natural one, which we denote by *N* ^*∗*^, is the number of vertices belonging to this cluster. *ϕ* is an order parameter associated with this choice as the relative size of that cluster:

(7) $$\begin{array}{c} \phi =\frac{N^{*}}{N}. \end{array}$$

The other choice is the number *L* ^*∗*^ of* k*-cliques of the largest* k*-clique percolation cluster. The associated order parameter is again the relative size of this cluster:

(8) $$\begin{array}{c} \varphi =\frac{L^{*}}{L}, \end{array}$$

where *L* denotes the total number of* k*-cliques in the graph. *L* can be estimated as

(9) $$\begin{array}{c} L\approx \left(\begin{array}{c} N\\ k \end{array}\right)d^{k\left(k-1\right)/2}\approx \frac{N^{k}}{k!}d^{k\left(k-1\right)/2}. \end{array}$$

In this paper, we use the biweight midcorrelation for constructing binary networks. Two-condition coexpression adjacency networks can always be transformed into a binary one by ignoring any directionality in the links and keeping only those stronger than a threshold weight. Then, the concept of maximum clique and* k*-clique algorithm were used to find gene differential coexpression modules. We named the proposed method “BMKC” (biweight midcorrelation and* k*-clique algorithm) method. Changing the threshold is like changing the resolution with which the community structure is investigated: by increasing, the communities start to shrink and fall apart. A very similar effect can be observed by changing the value of *k* as well: increasing *k* makes the communities smaller and more disintegrated but, at the same time, also more cohesive. More details about* k*-clique algorithm can be found in [28, 32].

## 3. Results 

### 3.1. Experiment Result on Simulated Datasets

We first evaluate the algorithm in a supervised setting. We generate a control group of 30 samples and a disease group of another 30 samples, both consisting of 120 genes. For the control group, 20 coexpressed genes are sampled directly from the biweight midcorrelation. We focus on whether* k*-clique algorithm can find coexpression gene modules from the background of noise. We first draw a vector with 20 rows and a vector with 30 columns from a standard normal distribution. The actual expression levels are obtained by adding independent errors sampled from a normal distribution with mean zero and standard deviation (SD) *σ*. These 20 genes form the target pattern. We then hide them in 100 additional noise genes, which are sampled independent and identically distributed (i.i.d.) from a standard normal distribution. The disease group is simulated by 120 independent noise genes drawn from a standard normal only.

**Figure 1:**
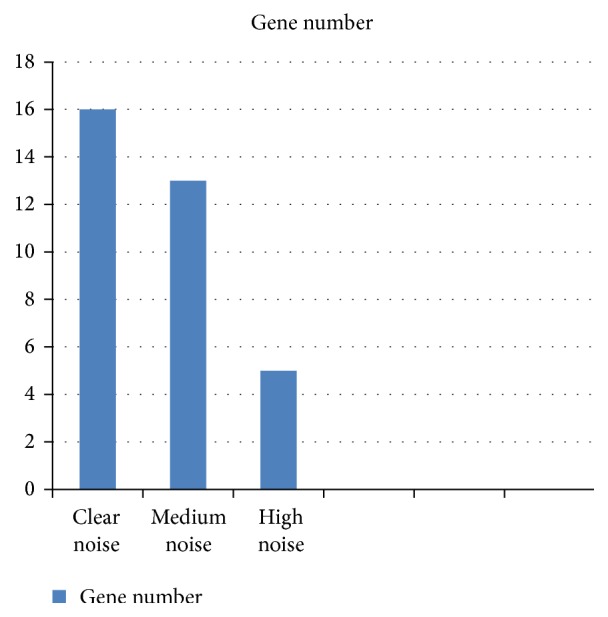
The column bar graph shows the effect of the noise parameter *σ* on the size of the gene group found by our algorithm.

In the above setting, we use SD *σ* to tune the strength of the signal resulting from the 20 coexpressed genes. To observe its effect in detail, we use three different values: for a clear signal, *σ* = 1/10, for medium noise, *σ* = 1/4, and, for high noise, *σ* = 1. To guard for sampling effects, we repeat each procedure 50 times and average the results, which are displayed in Figure 1. One can see that, for the clear and medium signal, the algorithm can recover the differentially coexpressed genes modules reliably. Also, depending on the prominence of the signal, the influence of *σ* is more or less pronounced. In an exploratory analysis setting with several hidden patterns, we could use *T*_1_, *T*_2_, and *T*_3_ to control the size of target patterns.

### 3.2. Analyzing a Type 2 Diabetes (T2D) in Rats

As a real-world application, we apply the BMKC method to a pair of type 2 diabetes (T2D) rats datasets (dataset pair *T*), which has been published in study [33]. Dataset pair *T* is from dataset GSE3068 of Gene Expression Omnibus (GEO) database. Yu et al. preprocessed dataset GSE3068. Dataset pair *T* includes 4765 genes in 10 disease samples and 10 normal samples. We use our algorithm to find differential coexpression modules in the type 2 diabetes.

**Figure 2:**
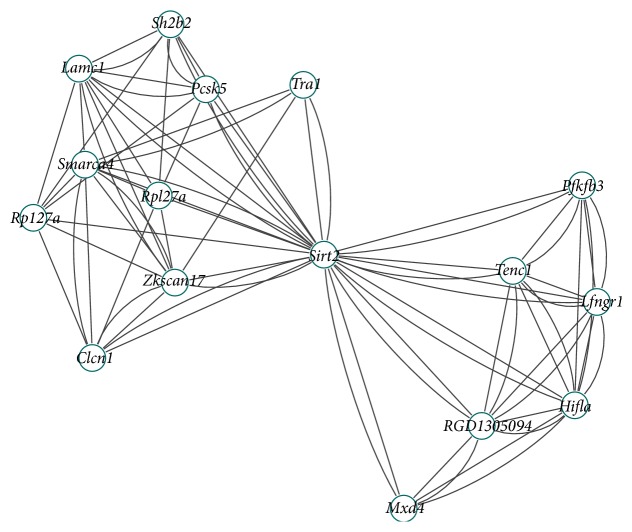
The adjacency graph of first gene differential coexpression module.

**Figure 3:**
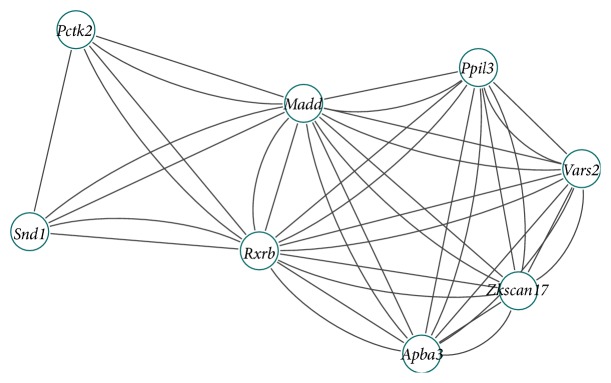
The adjacency graph of second gene differential coexpression module.

For computational efficiency, we calculate the sum of each row or column of adjacency matrix; the sum means the number of genes related to the gene. The gene is outlier if the sum is zero. First, we calculate the sum of each row or column of the adjacency matrix and delete the outlier. Second, we calculate the sum of each row or column of the adjacency matrix and discard the lower 50% of them. We set *T*_3_ = 1.3 and the minimum number of each clique to four. Finally, we apply our algorithm to the remainder genes and excavate two differential coexpression modules. Tables 1 and 2 list each gene symbol in the clique. The adjacency graphs of each differential coexpression module are shown in Figures 2 and 3. From these two figures, we can see that the cliques in each of the differential coexpression modules are overlapping, forming a closely related module. In normal condition, the absolute bicor value of total of 24 genes in modules distributes from 0.78 to 0.97. Yet, in disease condition, the absolute bicor value of genes distributes from 0.21 to 0.09. In the results of our study, the gene differential coexpression modules included quite a number of previously reported T2D-related genes:* Hifla* and* Sirt2* [34],* Smarca4* [35],* Sh2b2* [36],* Madd* [37], and* Rxrb* [38]. Despite not being previously reported to be related with T2D, other genes in the modules should receive adequate attention for their distinct traits from the perspective of differential coexpression. Further studies on the transcriptional mechanisms and functional consequences could pay more attention to these genes.

### 3.3. Significance Analysis of the BMKC Method

Naturally, the question of whether our findings are artifacts of the high dimensionality of the data arises. To assess this question, we apply a permutation procedure. Under the null hypothesis, we assume that all genes are mutually independent in both conditions groups. We heuristically sample from the null hypothesis by (group-wise) shuffling the expression values for each gene independently. Thus, random expression data are generated where all covariance structures are removed. Applying our algorithm to the randomized data yields one random score. We repeat the procedure 1000 times. Using the empirical distribution of the simulated scores, the simulated score means the global total sum of differential coexpression change of each gene in modules. We calculate *P* values for the observed scores in the nonpermuted data. For each of the patterns in the type 2 diabetes example, we only observe one random score smaller than the biological one. This corresponds to an empirical *P* value of 0.001. Hence, it is unlikely that the observed differential coexpression is a chance artifact.

## 4. Conclusions

In this paper, we proposed a new approach in gene sets level for differential coexpression analysis, which combine biweight midcorrelation and threshold selection strategy and also applied maximum clique concept with* k*-clique algorithm to the specific gene set to further investigate gene regulatory networks. Biweight midcorrelation is more robust for outliers and threshold selection strategy is an effective preprocess step of the proposed method. Experimental results on simulated datasets show that our method had good performance. We apply the proposed BMHT method to real dataset designed for T2D study, and two differential coexpression gene modules were detected, which should be a useful resource for T2D study and could be used for exploring the biological function of the related genes. In the future, we will focus on how to quickly excavate gene differential coexpression module from gene coexpression adjacency matrix.

## Figures and Tables

**Table 1:** Genes in each clique.

Clique number	Gene symbol				
1	***Hifla*** ^*^	*Ifngr1 *	*RGD1305094 *	*Tenc1 *	***Sirt2***
2	*Clcn1 *	***Smarca4***	*Zkscan17 *	*Rpl27a *	***Sirt2***
3	***Hifla***	*Ifngr1 *	*Pfkfb3 *	*Tenc1 *	***Sirt2***
4	***Sh2b2***	*Pcsk5 *	*Lamc1 *	*Rpl27a *	***Sirt2***
5	*Lamc1 *	***Smarca4***	*Zkscan17 *	***Sirt2***	*Rpl27a *
6	***Hifla***	*RGD130504 *	*Mxd4 *	***Sirt2***	
7	*Tra1 *	***Smarca4***	*Zkscan17 *	***Sirt2***	

^*∗*^Bold genes refer to the previously reported T2D-related genes. The other genes are identified in the differential coexpression modules.

**Table 2:** Genes in each clique.

Clique number	Gene symbol					
1	*Vars2 *	*Apba3 *	***Madd***	*Zkscan17 *	*Ppil3 *	***Rxrb***
2	*Snd1 *	***Madd***	*Pctk2 *	***Rxrb***		
